# Electrospun Polycaprolactone Nanofibers as a Reaction Membrane for Lateral Flow Assay

**DOI:** 10.3390/polym10121387

**Published:** 2018-12-14

**Authors:** Chee Hong Takahiro Yew, Pedram Azari, Jane Ru Choi, Farina Muhamad, Belinda Pingguan-Murphy

**Affiliations:** 1 Department of Biomedical Engineering, Faculty of Engineering, University of Malaya, Kuala Lumpur 50603, Malaysia; yewcheehongtakahiro@gmail.com (C.H.T.Y.); pedram.azari@gmail.com (P.A.); 2 Centre for Applied Biomechanics, Department of Biomedical Engineering, Faculty of Engineering, University of Malaya, Kuala Lumpur 50603, Malaysia; 3 Department of Mechanical Engineering, University of British Columbia, 2054-6250 Applied Science Lane, Vancouver, BC V6T 1Z4, Canada; janeruchoi@gmail.com; 4 Centre for Blood Research, Life Sciences Centre, University of British Columbia, 2350 Health Sciences Mall, Vancouver, BC V6T 1Z3, Canada

**Keywords:** electrospun PCL nanofibers, lateral flow assay, nucleic acid detection, point-of-care applications

## Abstract

Electrospun polycaprolactone (PCL) nanofibers have emerged as a promising material in diverse biomedical applications due to their various favorable features. However, their application in the field of biosensors such as point-of-care lateral flow assays (LFA) has not been investigated. The present study demonstrates the use of electrospun PCL nanofibers as a reaction membrane for LFA. Electrospun PCL nanofibers were treated with NaOH solution for different concentrations and durations to achieve a desirable flow rate and optimum detection sensitivity in nucleic acid-based LFA. It was observed that the concentration of NaOH does not affect the physical properties of nanofibers, including average fiber diameter, average pore size and porosity. However, interestingly, a significant reduction of the water contact angle was observed due to the generation of hydroxyl and carboxyl groups on the nanofibers, which increased their hydrophilicity. The optimally treated nanofibers were able to detect synthetic Zika viral DNA (as a model analyte) sensitively with a detection limit of 0.5 nM. Collectively, the benefits such as low-cost of fabrication, ease of modification, porous nanofibrous structures and tunability of flow rate make PCL nanofibers a versatile alternative to nitrocellulose membrane in LFA applications. This material offers tremendous potential for a broad range of point-of-care applications.

## 1. Introduction

Lateral flow assays (LFA) have been utilized for the rapid detection of biomarkers for various diseases and infections such as malaria, human immunodeficiency virus (HIV) and dengue virus [1,2,3,4]. Lateral flow test strips are fabricated by assembling components that are mainly made of cellulose and nitrocellulose, which include a sample pad, a reaction membrane, an absorbent pad and a supporting backing pad. They offer several advantages such as cost-effectiveness, simplicity of fabrication and ease of operation. The sample is added to the sample pad and the result can be observed by the naked eye within a few minutes. These benefits make it possible to apply them for point-of-care testing at rural areas, where laboratory facilities are often limited [5]. Recently, progress in the modification of test strips, such as incorporation of novel nanomaterials has improved the efficiency of LFA and satisfied the demands of diverse applications [6].

Electrospinning is a method to draw charged nanofibers from a polymer solution under a strong electric field [7]. The method has been advancing towards large-scale manufacture, production of organized structures through different strategies such as multiple-jet nozzle electrospinning [8] and needleless electrospinning [9], which have been employed to generate complex structure for biomedical functionalities, drug delivery systems and advanced composite nanofibers with fillers for biomimetic scaffolds [10,11,12,13,14,15]. Electrospun nanofibrous materials feature high surface-area-to-volume ratio, high porosity, interconnected porous network, and flexible functionality; characteristics that have been widely utilized in biomedical applications [16,17,18,19]. Despite having these favorable features, only a few studies have applied electrospun nanofibers in LFA. For example, electrospun cellulose nitrate has been integrated in electroresistive LFA for sensitive detection of *E. coli* O157:H7 [20]. Alternative reaction membrane of a colorimetric LFA with specific chemical features has been fabricated by electrospinning polylactic acid (PLA) with polyethylene glycol (PEG) and antifouling agent (polystyrene8K-block-poly(ethylene-ran-butylene)25K-block-polyisoprene10K-Brij76 (K3-Brij76 or KB)) to reduce non-specific binding of sulforhodamine B (SRB)-encapsulating liposomes during the detection of *E. coli* O157:H7 [21]. Alternatively, LFA was integrated with a bioresorbable electrospun scaffold in a theranostic system for the detection of wound biomarkers [22].

Among various types of electrospun nanofibers, aliphatic polyester nanofibers are generally bioresorbable, biocompatible, easily processible and non-toxic to cells. Therefore, they have been broadly used for tissue engineering, drug delivery and biosensor applications [23,24,25]. Polycaprolactone (PCL) electrospun nanofibers, as one of the aliphatic polyester nanofibers, has become a popular polymer for biomedical applications (e.g., development of biomedical devices) due to its slow degradation rate, relatively benign products of biodegradation and extraordinary physicochemical properties for chemical modifications [26]. The intrinsic hydrophobicity of electrospun PCL nanofibers acquires extra features, such as higher biocompatibility and hydrophilicity, through modifications like surface coating, plasma treatment, poly(dopamine) treatment, blending with a copolymer, alkali treatment and polymer grafting, which make it a favorable material for various biomedical applications [27]. For instance, Mao and Yoo increased the surface roughness of electrospun nanofibers by bleaching out pluronic after electrospinning the PCL/pluronic blend for better protein and cell adsorption [28]. Shahmoradi et al. has performed surface modification (i.e., alkaline hydrolysis) to induce the hydrophilicity and wettability of electrospun PCL nanofibers scaffold for effective cell growth [29]. Besides that, Asadian et al. has tuned the physical characteristics of nano-fibers (e.g., diameter and number of beads) by optimizing electrospinning parameters (e.g., voltage, feed rate, electro-spinning distance and PCL concentration) to improve protein adsorption as well as cell attachment and cell spreading [30]. In another study, Guler et al. has performed electrochemical detection of single-stranded DNA with electroactive polypyrrole-coated electrospun PCL nanofibers that had high loading capacity for bioactive molecules due to a high surface area to volume ratio [23]. Despite the tremendous potential of electrospun PCL nanofibers as an alternative material for diverse biomedical applications owing to their exceptional properties, their potential application in LFA has not been explored yet.

This study demonstrates the application of electrospun PCL nanofibers as a reaction membrane for LFA for the first time. The nanofibers were subjected to alkaline hydrolysis in different conditions, including a range of alkaline concentration and duration of hydrolysis to achieve the optimal conditions of treatment in terms of hydrophilicity and wettability. The PCL nanofibrous membranes were further characterized via several analytical methods such as field emission scanning electron microscopy (FESEM), water contact angle, porosity and wicking time measurements. The hydrolyzed electrospun PCL nanofibers were then implemented in LFA strips for the detection of nucleic acid (Zika viral cDNA as a model analyte). With the optimized NaOH treatment conditions, the proposed material was able to detect Zika viral DNA sensitively with a detection limit as low as 0.5 nM. Taken together, the proposed electrospun PCL nanofibers show significant promise to be an alternative reaction membrane for LFA to achieve optimum device performance at minimal cost.

## 2. Materials and Methods

### 2.1. Fabrication of Electrospun Polycaprolactone (PCL) Nanofibers

**Figure 1:**
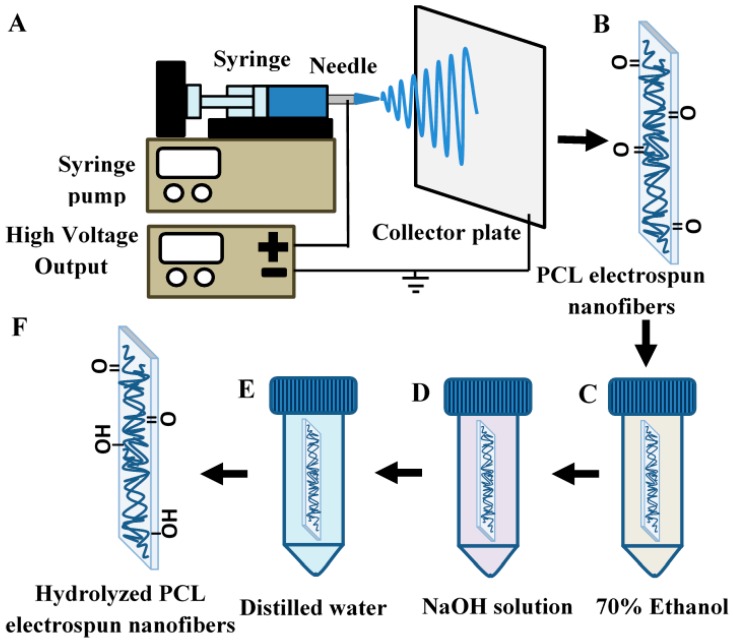
Schematic of electrospinning and alkaline hydrolysis of polycaprolactone (PCL). PCL nanofiber is fabricated using an electrospinning setup (**A**); The electrospun PCL nanofiber membrane (**B**) is soaked in 70% ethanol (*v*/*v* distilled water) (**C**) for a more thorough alkaline hydrolysis in NaOH solution (**D**); The treated electrospun PCL nanofiber membrane is then rinsed in distilled water (**E**) and dried to acquire the resultant hydrolyzed electrospun PCL nanofiber membrane (**F**).

PCL (Mn = 80,000; Sigma Aldrich, Saint Louis, MI, USA) was dissolved in a co-solvent of 9:1 (*v*/*v*) Chloroform (Friendemann Schmidt, Parkwood, Australia): DMF (Merck, Darmstadt, Germany) and stirred at room temperature to obtain the electrospinning dope (10% *w*/*v*). The 20 mL electrospinning dope was then electrospun using an electrostatic field of 12 kV (Gamma High Voltage Research, Ormond Beach, FL, USA), a blunt 20 G needle (Terumo, Laguna, Philippines) and an aluminum collector placed 18 cm from the needle horizontally. The feeding rate was kept constant at 3 mL/h via a KDS 100 syringe pump (KD Scientific, Inc., Holliston, MA, USA). The electrospun PCL nanofibrous membrane was then left to dry overnight in 37 °C. The setup for electrospinning is depicted in Figure 1A.

### 2.2. Alkaline Hydrolysis of Electrospun PCL Nanofibers

Electrospun PCL nanofibrous membranes (Figure 1B) were initially immersed in 70% ethanol (*v*/*v* water; Systerm Chemicals, Shah Alam, Malaysia) for 15 min (Figure 1C) before soaking in different concentrations (1 mM–2 M) of sodium hydroxide (NaOH; Merck, Germany) aqueous solution for various durations (1–2 h) (Figure 1D) under shaking conditions at room temperature. After the alkaline treatment, the NaOH solution on the nanofibrous membranes was then rinsed off thoroughly with distilled water (Figure 1E) and dried at 37 °C. Alkaline treatment beyond 2 M was not carried out as a higher concentration of NaOH solution would lead to intense degradation of PCL nanofibrous membrane and inhibit further processing of the sample, as shown in Supplementary Figure S1.

### 2.3. Morphology Characterization

The dry hydrolyzed PCL and non-hydrolyzed PCL nanofibrous membranes were sputtered with gold before the morphologies were examined by FESEM (Quanta FEG 650, Thermo Fisher Scientific, Waltham, MA, USA). Fiber diameter and pore size distributions were analyzed using Fiji software [31]. A minimum of 40 measurements were conducted for each sample to analyze fiber diameter and pores.

### 2.4. Porosity Estimation

The porosities of hydrolyzed PCL and PCL nanofibrous membranes were estimated using a pycnometer (Marienfeld, Germany) filled with absolute ethanol (John Kollin Corporation, Midlothian, United Kingdom). The porosity based on Archimedes’ principle was calculated as the following [32]:

(1) $$\mathrm{Porosity}\;\left(\%\right)=\frac{W_{2}-W_{3}-W_{m}}{\rho_{e}}/\frac{W_{1}-W_{3}}{\rho_{e}}\times 100$$

where *W*_1_ is the weight of the pycnometer filled with absolute ethanol, *W*_2_ is the weight of pycnometer filled with absolute ethanol and nanofibrous membrane, *W*_3_ is the weight of pycnometer and absolute ethanol when the ethanol-soaked nanofibrous membrane had been taken out from *W*_2_, *W*_m_ is the dry weight of the nanofibrous membrane, and *ρ*_e_ is the density of the absolute ethanol.

### 2.5. Water Contact Angle

A 10 μL droplet of distilled water was dispensed onto the PCL nanofibrous membrane and hydrolyzed electrospun PCL nanofibrous membrane using a micropipette. A OnePlus 3T smartphone (Oneplus, Hong Kong, China) was used to record the droplet dispensing process at 4 K resolution with external lighting. VLC media player (VideoLan Organization, Paris, France) was used to obtain a zoom-in snapshot of the droplet from the recorded video at one second after dispensing the droplet on the membrane surface. The static water contact angle of the droplet was then analyzed using DropSnake plug-in in the Fiji software [33].

### 2.6. Preparation of Gold Nanoparticles (AuNPs) and AuNP-Detecting Probe Conjugates (AuNP–DPs)

Gold nanoparticles (AuNPs) of 13 nm diameter was prepared as reported in previous work [24]. Before conjugation, detecting probe (DP; Bio Basic Inc., Ontario, Canada; sequence: 5′-SH-C6-ATC ATC GA AGT GGC TTC A-3′) was prepared by adding 500 mM acetate buffer (pH 4.68, which consist of 1 M sodium acetate (Ajax Finechem Pty. Ltd., NSW, Australia) and 0.21 M acetic acid (Fisher Scientific) to the lyophilized DP and topped up to 100 μM with distilled water. AuNP–DP conjugates were prepared by adding 6 μL of 100 μM detecting probe (DP) to 1 mL AuNPs solution and left for 16 h for conjugation. 1% sodium dodecyl sulfate (SDS) (Sigma Aldrich) and 2 M NaCl (Merck) was then added to the solution to achieve a final concentration of 0.0% SDS and 0.16 M NaCl in the solution. The solution was then allowed to age for at least 24 h before centrifugation at 14,000 g for 25 min. Following centrifugation, the supernatant was removed while the pellet was resuspended in 67 μL eluent buffer, which comprised of 5% bovine serum albumin (*w*/*v*; AMRESCO, Solon, OH, USA), 0.25% Tween-20 (*w*/*v*; Sigma Aldrich), 10% sucrose (*w*/*v*; Ajax Finechem Pty. Ltd.) and 20 nM trisodium phosphate (Sigma Aldrich). The resulting AuNP–DP conjugate solution was kept at 4 °C for future use. We have characterized the AuNPs in our previous publication. Their average sizes are 13 nm [3].

### 2.7. Fabrication of Lateral Flow Assay (LFA) Strip

**Figure 2:**
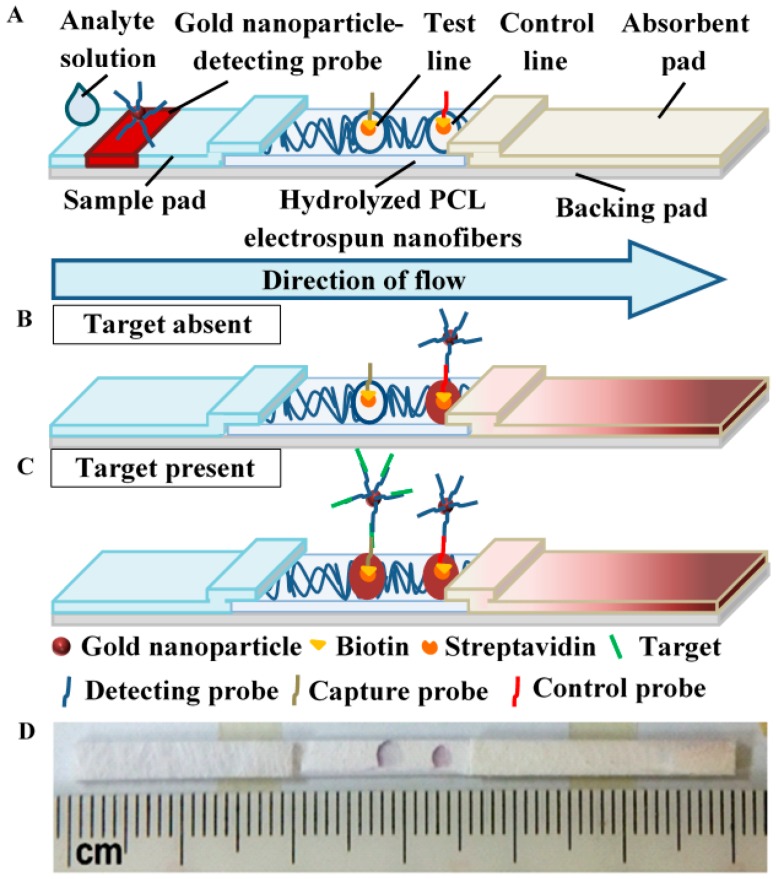
Schematic of lateral flow assay (LFA) with hydrolyzed electrospun PCL nanofibers for DNA detection. The common LFA strip was assembled with the hydrolyzed electrospun PCL nanofibers (**A**); The analyte solution would flow from the sample pad to the backing pad through the hydrolyzed membrane. In the absence of target analyte, the assay would not show a signal on the test line (**B**); In the presence of target analyte, the target would bind to the detecting probe and capture probe and produce a positive signal on the test line (**C**); A representative image of the test strip made of electrospun PCL nanofibers (**D**).

Hydrolyzed electrospun PCL nanofibrous membrane (2.0 cm × 0.25 cm), Pall 8964 glass fibers sample pad (2.0 cm × 0.25 cm × 0.05 cm; Pall Corporation, Port Washington, NY, USA) and H-1 absorbent pad (2.5 cm × 0.25 cm × 0.1 cm; JNBio Co., Ltd., Guangzhou, China) were tailored using a guillotine paper cutter before their assembly by mounting on a piece of J-B6 backing card (6.0 cm × 0.25 cm × 0.02 cm; JNBio Co., Ltd.), as shown in Figure 2A. The sample pad was treated with 0.1% of Tween-20 and dried in the oven before assembly. After the assembly, 6 μL of AuNP–DPs were added to the middle of the sample pad while 0.5 μL of control probes and capture probes were dispensed onto hydrolyzed PCL using a micropipette to produce a control line and a test line, respectively. The test strip was then dried overnight at 37 °C before use. Prior to dispensing, the lyophilized control probe (Bio Basic Inc.; sequence: 5′-T GAA GCC ACT GTG AGA-BIOTIN-3′) and capture probes (Bio Basic Inc.; sequence: 5′-AAT GCT TTT CCG CC-BIOTIN-3′) were prepared by adding 2 mg/mL streptavidin (Promega, Madison, WI, USA)-phosphate buffered saline (PBS) (Sigma Aldrich) solution and 10 mM PBS to dissolve the probes. The probes were then left for 1 h before topping up to 100 mM with absolute ethanol.

### 2.8. Wicking Time Measurement

LFA strips with hydrolyzed PCL nanofibrous membrane were dipped vertically into wells of 96 well plates each containing 80 μL SSC×4 solution (Roche Diagnostics GmbH, Mannheim, Germany). The time taken for the lateral flow to travel from the connecting point of sample pad and hydrolyzed PCL to the test line was measured using a CT-20 digital timer (Canon, Tokyo, Japan), respectively.

### 2.9. Colorimetric Analysis of LFA

Prior to the assay, various concentrations (0.025–10 nM) of analyte solution were prepared by serial dilution after dissolving lyophilized target single-stranded DNA (ssDNA; Bio Basic Inc.; sequence: 5′-GGA AAA GCA TTT GAA GCC ACT GTG AGA-3′) in SSC×4 solution. The details of the target ssDNA, detecting probe, capture probe and control probe can be referred in Supplementary Table S1. LFA strips with hydrolyzed electrospun PCL nanofibers were dipped vertically into the wells of 96 well plates containing 80 μL analyte solution. As the LFA strips were dipped in the analyte solution, the present target ssDNA strands are brought up by capillary effect in the glass fibers to bind the AuNP-DPs via DNA-DNA hybridization. As the analyte solution continues to wick towards the absorbent pad, the AuNP–DP-bound target ssDNA strands hybridize with the capture probe ssDNA on the reaction membrane, forming a red signal at the test line. The assay is depicted in Figure 2B,C when the target was absent and present in the analyte solution, respectively. Once the sample had completely wicked through the strips, shots of the resultant LFA strips were taken using a FinePix F665EXR camera (Fujifilm, Tokyo, Japan). The mean optical densities of the test line (*OD*_T_) and background (*OD*_Bg_) on the hydrolyzed PCL reaction membrane were measured using Image Pro Plus software (Media Cybernetics. Inc., Bethesda, MD, USA). The signal-to-noise ratio (SNR) of LFA strips with hydrolyzed PCL were compared. The SNR of LFA was determined as the following [34]:

(2) $$\mathrm{SNR}=\left(\frac{OD_{T\left[c\right]}}{OD_{\mathrm{Bg}\left[c\right]}}\right)/\left(\frac{OD_{T\left[0\right]}}{OD_{\mathrm{Bg}\left[0\right]}}\right)$$

where *c* was the concentrations of the target ssDNA and 0 was 0 M target ssDNA in the analyte, respectively. A completed assay strip is showed in Figure 2D.

### 2.10. Statistical Analysis

Data were tested by one-way analysis of variance (ANOVA) with Tukey’s post hoc test using IBM SPSS Statistics 24 software. Data are represented as mean ± standard error of the mean of at least 3 independent experiments. *p* < 0.05 was reported as statistically significant.

## 3. Results and Discussion

### 3.1. Hydrolysis of Electrospun PCL Nanofibers as Reaction Membrane

Some essential properties of paper-based materials used in lateral flow assay (LFA) strips are pore size, surface area-to-volume ratio and chemical nature that governs functionalization for fluidic manipulation and non-specific reaction [21]. Commercial nitrocellulose has been commonly employed as a reaction membrane due to its sufficiently large pores and chemistry for high-level protein adsorption and wetting. However, modifications are usually required to achieve desirable flow rate and optimum signal detection. In a similar manner, electrospinning has the flexibility to finetune the reaction membrane with the desired properties through controlling processing and environmental parameters such as applied voltage, pumping speed and relative humidity. Yet, as a hydrophobic material, electrospun PCL nanofibers is not feasible in wetting and wicking. To enable fluidic control and produce the optimal colorimetric signal on an LFA strip, further chemical modification of the nanofibers is required. In this study, PCL was electrospun and further processed in a series of chemical soaking, i.e., 70% ethanol, NaOH and distilled water before it is dried for use (Figure 1). The alkaline hydrolysis was performed to develop hydrophilicity in reaction membranes for improved fluid flow. This step functionalizes the polyester with carboxyl and hydroxyl groups on the membrane, which also facilitates protein-binding reaction [35,36]. Here, the resulting hydrolyzed electrospun PCL nanofibers were installed into LFA strips to replace the commonly used nitrocellulose membrane (Figure 2A). Optimal flow rate was obtainable by determining the right concentration of NaOH used in the alkaline hydrolysis process. The ease of fabrication and modification to achieve desirable functionality of reaction membrane has made PCL a promising alternative material for LFA.

### 3.2. Surface Morphology

**Figure 3:**
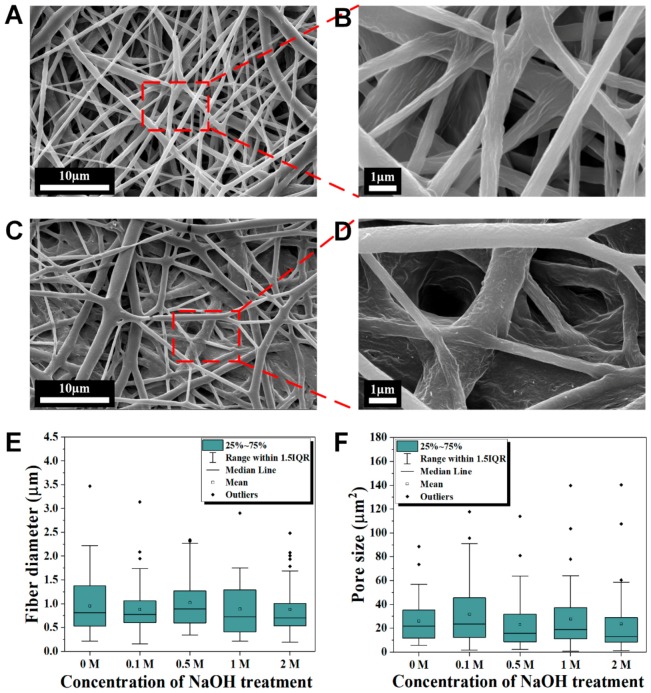
Surface morphology of PCL and hydrolyzed electrospun nanofibers. Compared to electrospun PCL nanofibers (**A**,**B**), hydrolyzed electrospun PCL nanofibers (**C**,**D**) after 1.5 h of 2 M NaOH treatment shows slight degradation at the external fibers while flattening and denser arrangement in the internal. The fiber diameter (**E**,**F**) pore size distributions of the electrospun PCL nanofibers at different concentrations of NaOH treatment shows no significant differences at the mean values (*p* > 0.05), *n* = 47 for each concentration in (**E**,**F**), respectively.

To evaluate the effect of alkaline hydrolysis on the physical morphology of electrospun PCL nanofibrous membranes, microstructures of the unhydrolyzed and hydrolyzed membranes were examined with FESEM (Figure 3 and Supplementary Figure S2). Fibrous and porous structures can be observed in both electrospun PCL nanofibers (Figure 3A,B) and 2M NaOH hydrolyzed electrospun PCL nanofibers (Figure 3C,D). Compared to electrospun PCL nanofibers, the hydrolyzed membrane had flatter fibers that appeared to be more densely packed. Furthermore, slight erosion of fibers may have happened when subject to NaOH at a high concentration as the surface of the nanofibers became rougher. However, there is no significant difference (*p* > 0.05) in average fiber diameter and pore size between the electrospun PCL nanofibers and membranes hydrolyzed with various concentrations of NaOH, as shown in the distribution boxplots (Figure 3E,F). Meanwhile, the duration (1, 1.5 and 2 h) of alkaline hydrolysis also did not show any significant difference (*p* > 0.05) in the average surface fiber diameter and pore size of the PCL electrospun nanofibers (Supplementary Figure S2G,H). It is noteworthy that the hydrolyzed nanofibers without 70% ethanol soaking step before the NaOH treatment were less compact and flattened (Figure S3). The 70% ethanol soaking step had facilitated the diffusion of NaOH solution within the membrane, extending the hydrolysis reaction to the internal layers of nanofibers. There is also a significant reduction of average thickness of the electrospun nanofibers membrane at 2 M NaOH (Supplementary Figure S4), which is the result of the peeling effect suggested to happen at prolonged hydrolysis by higher concentrations of alkaline [37]. This effect is related to exposition and release of fragments of degradation products into the solution [38].

### 3.3. Wettability and Wickability Analysis of PCL and Hydrolyzed Electrospun PCL Nanofibrous Membrane

**Figure 4:**
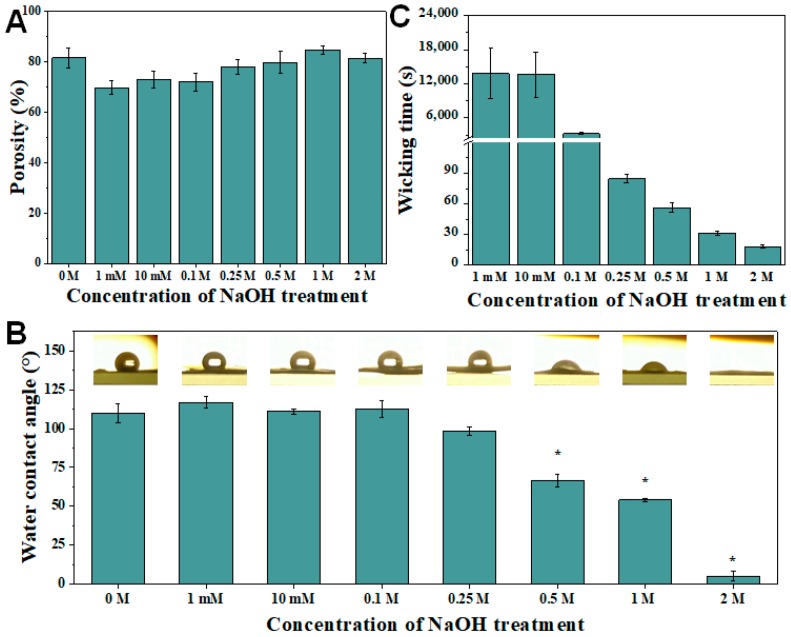
The influence of NaOH concentration upon wetting and wicking parameters. The porosity (**A**) of the post-treated nanofibers was not significantly affected by the NaOH concentration. The water contact angle shows a significant reduction at NaOH concentration lower than 0.25 M (**B**); The wicking time of liquid from sample pad to test line of NaOH-treated electrospun PCL nanofibers substantially reduced as the NaOH concentration increased at 0.25 M (**C**). *n* = 4 for each concentration in (**A**,**B**), and *n* = 3 for each concentration in (**C**). * represents *p* < 0.05.

To study the effect of alkaline hydrolysis on the pore distribution in the electrospun PCL nanofibrous membrane, the porosity of the treated membranes was analyzed. The porosity of the electrospun PCL nanofibrous membranes hydrolyzed with 0 M (no treatment), 1 mM, 10 mM, 0.1 M, 0.5 M, 0.25 M, 1 M and 2 M NaOH were 81.58% ± 3.97%, 69.81% ± 2.77%, 72.89% ± 3.33%, 72.08% ± 3.57%, 78.09% ± 5.07%, 79.89% ± 4.24%, 84.77% ± 1.66% and 81.41% ± 1.83%, respectively, which showed no significant difference (*p* > 0.05) between the treated and untreated nanofibrous membrane (Figure 4A). Meanwhile, the porosity of the electrospun PCL nanofibrous membranes hydrolyzed with 1 M NaOH at 0 h (no treatment), 1, 1.5 and 2 h were 81.58% ± 3.97%, 85.27% ± 3.05%, 84.78% ± 2.89% and 88.75% ± 3.10%, respectively (Figure S5A). Similarly, the differences of porosities for 0–2 h durations of 1 M NaOH were also not significant (*p* > 0.05). In the pore size and fiber diameter analyses, the alkaline concentration (1 mM–2 M) and treatment duration (1–2 h) did not have a significant effect on the physical properties of the pores in the nanofibrous membrane.

To evaluate the wettability of electrospun PCL nanofibrous membrane after alkaline hydrolysis, static water contact angles of a water droplet on the hydrolyzed membranes were measured. The contact angle of the water droplet on the hydrolyzed electrospun PCL nanofibrous membrane decreases significantly as the NaOH concentration exceeds 0.5 M (Figure 4B). At higher NaOH concentrations, more OH^−^ ions catalyze the hydrolysis of ester linkage in the PCL polymer, leading to the formation of more hydroxyl (OH) and carboxylic (COOH), which enables the hydrolyzed PCL to form hydrogen bonds with water molecules [29]. The alkaline hydrolysis of an ester can be described as the following chemical equation:

$$\mathrm{RCOOR}\prime \overset{{\mathrm{OH}}^{-}}{\to }\mathrm{RCOOH}+\mathrm{HOR}\prime$$

The higher affinity to water attributes to the enhanced hydrophilicity and wettability of the hydrolyzed electrospun PCL nanofibers that is exhibited by the decreasing water contact angle. Meanwhile, there was no significant difference in water contact angle (*p* > 0.05) between the durations of NaOH treatment (Supplementary Figure S5B).

To investigate the wickability of the hydrolyzed electrospun PCL nanofibrous membrane, the wicking time of SSC×4 running buffer to the test line (T-line) on the LFA strip with hydrolyzed electrospun PCL nanofiber membrane was recorded. Due to the hydrophobic nature of electrospun PCL nanofibers, wicking is virtually impossible unless it is modified. Here, the alkaline hydrolysis by NaOH treatment increased the hydrophilicity of electrospun PCL nanofibers and hence induced wickability. Furthermore, the increase in NaOH concentration reduced the wicking time of SSC×4 running buffer to T-line (Figure 4C). The decreasing water contact angle of water droplets on the nanofibers represents the increase in penetrability, which results in a reduction of wicking time of liquid [39]. Due to the limited hydrolysis at very low NaOH concentrations (≤0.1 M), there was a long delay in wicking time (>45 min) (Supplementary Figure S6A). Hence, NaOH concentration of at least 0.25 M was chosen for the following sensitivity test as to fulfill a fast and efficient assay. On the other hand, there is no significant difference on the wicking time between different durations of 1 M NaOH treatment (Supplementary Figure S5C). In addition, it is worthy of note that the wicking on hydrolyzed electrospun PCL nanofibrous membrane without 70% ethanol soaking was only partial and did not wick through the T-line and C-line as the hydrolysis only happened at the superficial level (Supplementary Figure S6B).

### 3.4. Detection of Nucleic Acid in LFA

**Figure 5:**
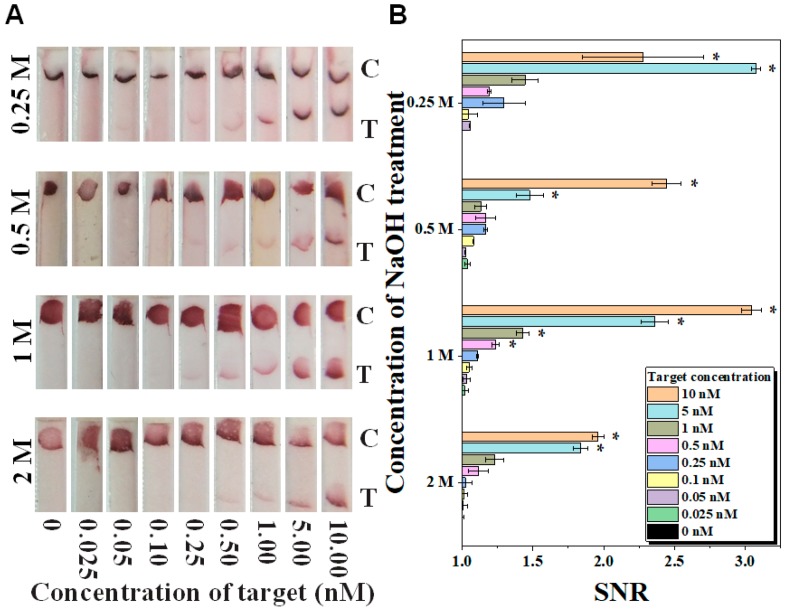
The effect of concentration of NaOH treatment on the performance of lateral flow assay (LFA) integrated with hydrolyzed electrospun PCL nanofibers for the detection of zika viral DNA. Qualitatively, the background or noise signals were stronger at test strips with electrospun PCL membrane treated with NaOH concentration beyond 1 M (**A**); The quantitative results for signal-to-noise ratio (SNR) showed the highest LOD at 0.5 nM for LFA with hydrolyzed electrospun PCL nanofibrous membrane treated with 1 M NaOH (**B**). *n* = 3. * represents *p* < 0.05.

To demonstrate the potential of hydrolyzed electrospun PCL nanofibers for the detection of biological molecules, LFA were performed for the detection of synthetic zika viral DNA (as a model analyte) at a range of 0.025–10 nM. The tests were set up with the incorporation of electrospun PCL nanofibrous membrane treated with various NaOH concentrations (0.25, 0.5, 1 and 2 M) and durations (1, 1.5, and 2 h). It was observed that the SNR of the visual signal on the LFA strips increased with increasing target concentrations. This is because more target molecules are bound at the T-line on each LFA strip with electrospun PCL nanofibers hydrolyzed with NaOH. Qualitatively, along with the increased concentration of targets, the color intensity of the T-line increased (Figure 5A). Based on the data of quantitative analysis, the limits of detection (LOD) were 5 nM for 0.25 M, 0.5 M and 2 M NaOH treated electrospun PCL nanofibers, and 0.5 nM for 1 M NaOH treated PCL electrospun nanofibers (Figure 5B). Notably, the strips with electrospun PCL nanofibers treated with 0.5 M and 0.25 M NaOH have higher background signal compared to the ones with 1 M NaOH treatment. In other words, 1 M NaOH treatment on electrospun PCL nanofibers has enabled the best visual detection on LFA as a strong signal is developed with minimal background signal. On a different duration of 1 M NaOH treatment, it was observed that the color intensity of the T-line increased along with the increased concentration of targets (Supplementary Figure S7A). For quantitative analysis, the LODs were 1 nM for 1 h, 0.5 nM for 1.5 h and 5 nM for 2 h treatment (Supplementary Figure S7B). Taken together, 1.5 h of 1 M NaOH treatment showed the strongest signal intensity with relatively low background signal. Hence, hydrolyzed electrospun PCL nanofibers with 1.5 h of 1 M NaOH treatment should be used during LFA for DNA detection.

In short, the fabrication of hydrolyzed electrospun PCL nanofibers and their potential application in LFA for the detection of nucleic acids were demonstrated. By alkaline hydrolysis, the electrospun PCL nanofibers were modified to become more hydrophilic and wickable to achieve the desirable functionality of reaction membrane. In addition, the benefits such as low cost of fabrication, ease of modification, porous nanofibrous structures, tunability of flow rate through controlling the concentration of the alkaline solution make PCL a versatile alternative to nitrocellulose membrane in LFA applications. A comparison between current work and other reported electrospun materials for a lateral flow biosensor was summarized in Table 1. As compared to electrospun polylactic acid nanofibers with the infusion of expensive biotin protein, hydrolyzed electrospun PCL nanofibers only require low-cost modification, i.e., NaOH treatment [40]. In contrast to the co-electrospun nanofibers of PLA–PEG–KB, there is no extra anti-fouling agent required in hydrolyzed electrospun PCL nanofibers to eliminate non-specific binding as the optimum NaOH concentration and duration of treatment would achieve the desirable low background signal [21]. Direct probe immobilization on hydrolyzed electrospun PCL nanofibers was possible without cross-linking agent such as glutaraldehyde in electrospun nitrocellulose [41]. Future work should include the optimization of hydrolysis with various alkalis, e.g., potassium hydroxide, ammonia, etc., and application using real samples (e.g., detecting targets in blood or food samples). We envision the hydrolyzed electrospun PCL nanofibers as a promising platform in point-of-care diagnostics.

## 4. Conclusions

The present study has successfully fabricated an alternative reaction membrane for LFA by thorough hydrolysis of electrospun PCL nanofibers with NaOH treatment to introduce supporting features of lateral flow. The concentration and duration of NaOH treatment did not influence the physical properties of nanofibers, i.e., average fiber diameter, average pore size and porosity. However, a significant reduction of the water contact angle was observed due to generation of hydroxyl and carboxylic groups on the nanofibers at increasing NaOH concentration. The wickability of hydrolyzed electrospun PCL nanofibers was significantly enhanced for LFA application when the sample was treated with 1 M NaOH for 1.5 h. With these optimal conditions, the hydrolyzed electrospun PCL nanofibers-incorporated LFA enables the detection of ssDNA with a LOD of 0.5 nM at minimal background signal. In addition to the wickability, the possibility of specific protein binding due to the generation of hydrophilic groups on the hydrolyzed electrospun PCL nanofibers possess great potential for the detection of myriad target analytes at point-of-care in the near future.

## Figures and Tables

**Table 1:** Characteristics of the current hydrolyzed electrospun PCL nanofibers and existing electrospun materials in lateral flow sensing application.

Material	Characteristics	Reference
Electrospun polylactic acid nanofiber infused with biotin	Simple setup Rapid detection High background signal Low signal intensity High cost of biotin	[40]
Plasma-treated electrospun nitrocellulose	Low cost Require glutaraldehyde for antibody attachment Instrument-dependent for signal observation Complicated setup	[41]
Co-electrospun nanofibers of poly(lactic acid), poly(ethylene glycol) and polystyrene8K-block-poly(ethyleneran-butylene)25K-block-polyisoprene10K-Brij76	Simple setup Uneven test and control lines Anti-fouling agent	[21]
NaOH-hydrolyzed electrospun polycaprolactone nanofibers	Low cost Low background signal Ease of fabrication and modification	This work

## Supplementary Materials

The following are available online at http://www.mdpi.com/2073-4360/10/12/1387/s1, Figure S1: Effect of extremely high NaOH concentration on electrospun PCL nanofibrous membrane, Figure S2: Surface morphology of PCL and hydrolyzed electrospun nanofibers at different concentrations and durations, Figure S3: Effect of 70% ethanol soaking on the surface morphology of hydrolyzed electrospun PCL nanofibers, Figure S4: Thickness of the electrospun PCL nanofibers membrane after treatment of NaOH, Figure S5: The influence of NaOH treatment duration upon wetting and wicking parameters, Figure S6: Wicking properties of the test strips with unoptimized hydrolyzed electrospun PCL nanofibers, Figure S7: The effect of duration of NaOH treatment on the performance of lateral flow assay (LFA) integrated with hydrolyzed electrospun PCL nanofibers for the detection of zika viral DNA, Figure S8: ATR-FTIR absorbance spectra of electrospun PCL plotted against electrospun PCL treated with 2 M NaOH solution for 1.5 h, Table S1: Details of the DNA probes and target DNA.
