# Supplementary material for: Electrospun Polycaprolactone Nanofibers as a Reaction Membrane for Lateral Flow Assay
Source: Polymers (Basel). 2018 Dec 14;10(12):1387. doi: 10.3390/polym10121387 (PMC6401928; doi:10.3390/polym10121387)
Supplement: Supplementary file 1 [file polymers-10-01387-s001.pdf]

polymers

MDPI

Article

Polycaprolactone Electrospun Nanofibers as a Reaction Membrane for Lateral Flow Assay

Chee Hong Yew [1], Pedram Azari [1,2], Jane Ru Choi [3,4], Farina Muhammad [1] and Belinda Pingguan Murphy [1,*]

[1] Department of Biomedical Engineering, Faculty of Engineering, University of Malaya, 50603 Kuala Lumpur, Malaysia
[2] Centre for Applied Biomechanics, Department of Biomedical Engineering, Faculty of Engineering, University of Malaya, 50603 Kuala Lumpur, Malaysia
[3] Department of Mechanical Engineering, University of British Columbia, 2054-6250 Applied Science Lane, Vancouver BC V6T 1Z4, Canada
[4] Centre for Blood Research, Life Sciences Centre, University of British Columbia, 2350 Health Sciences Mall, Vancouver BC, V6T 1Z3, Canada
* Correspondence: bpingguan@um.edu.my; Tel.: +60-3-7967-4491

Received: date; Accepted: date; Published: date

Abstract: Polycaprolactone (PCL) electrospun nanofibers have emerged as a promising material in diverse biomedical applications due to their various favorable features. However, their application in the field of biosensors such as point-of-care lateral flow assays (LFA) has not been investigated. The present study demonstrates the use of PCL electrospun nanofibers as a reaction membrane for LFA. PCL electrospun nanofibers were treated with NaOH solution for different concentration and duration to achieve a desirable flow rate and optimum detection sensitivity in nucleic acid-based LFA. It was observed that the concentration of NaOH does not affect the physical properties of nanofibers, including average fiber diameter, average pore size and porosity. However, interestingly, a significant reduction of the water contact angle was observed due to generation of hydroxyl and carboxyl groups on the nanofibers, which increased their hydrophilicity. The optimally treated nanofibers were able to sensitively detect synthetic Zika viral DNA (as a model analyte) with a detection limit of 0.5 nM. Collectively, the benefits such as low-cost of fabrication, ease of modification, porous nanofibrous structures and tunability of flow rate make PCL a versatile alternative to nitrocellulose membrane in LFA applications. This material offers tremendous potential for a broad range of point-of-care applications.

Keywords: polycaprolactone, electrospun nanofibers, lateral flow assay, nucleic acid detection, point-of-care applications

Supplementary materials

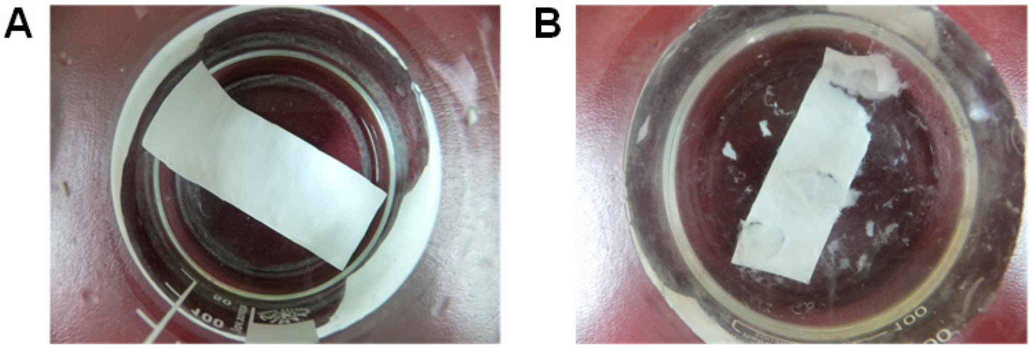

Figure S1. Effect of extremely high NaOH concentration on PCL electrospun nanofibrous membrane. Hydrolyzed PCL electro-spun nanofibrous membrane by 2 M NaOH physically showed no changes during rinsing at post-treatment (A). The membrane after 3 M NaOH treatment was highly degraded and broken into pieces during handling and rinsing with distilled water (B).

Table S1. Details of the DNA probes and target DNA

Name	Size (bp)	Sequence
Detecting probe	18	5'-SH-C6-ATC ATC GA AGT GGC TTC A-3'
Control probe	16	5' -T GAA GCC ACT GTG AGA-BIOTIN-3'
Capture probe	14	5'-AAT GCT TTT CCG CC-BIOTIN-3'
Target	27	5'-GGA AAA GCA TTT GAA GCC ACT GTG AGA-3'

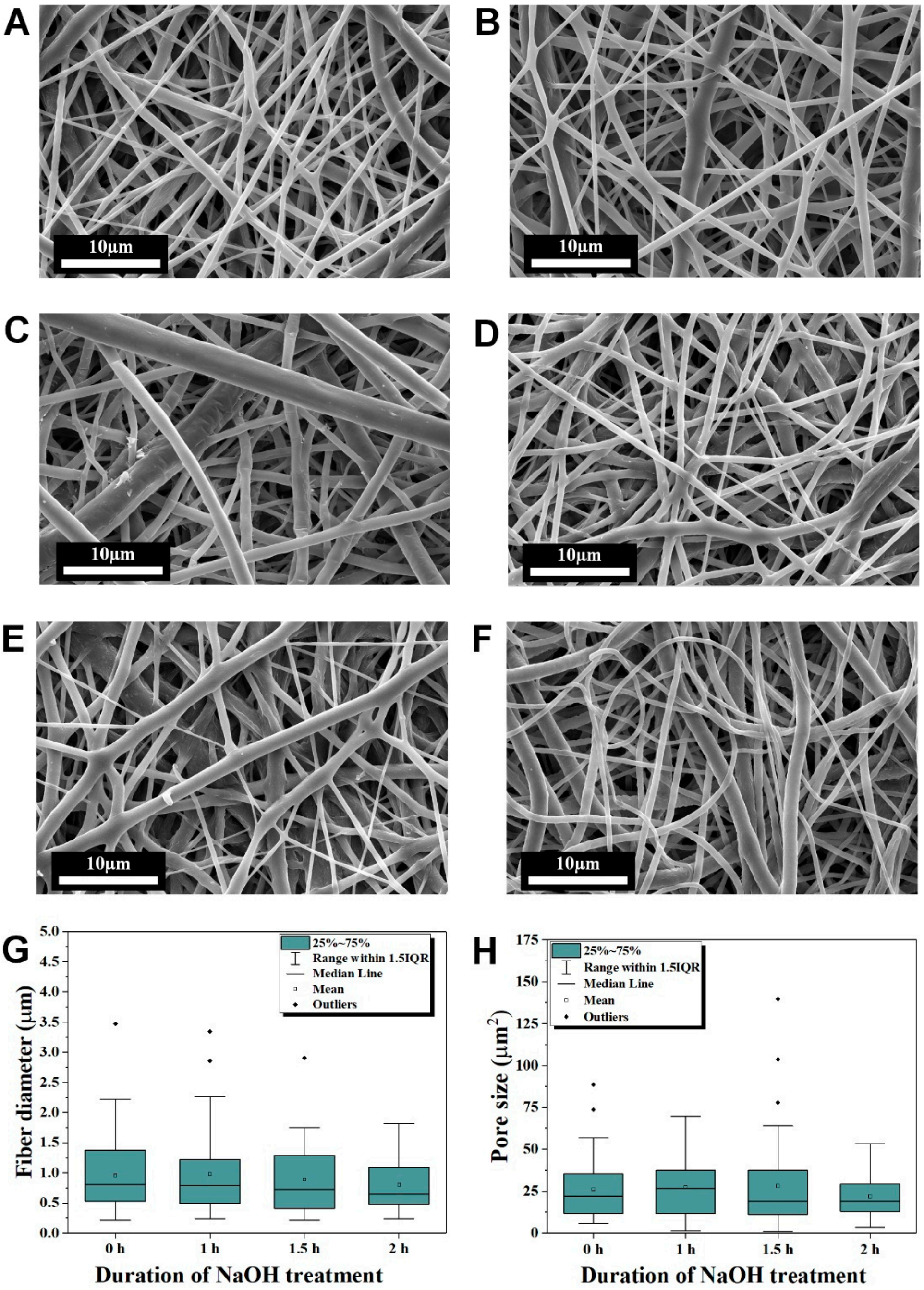

Figure S2. Surface morphology of PCL and hydrolyzed electrospun nanofibers at different concentrations and durations. The FESEM images showed an insignificantly gradual flattening of PCL electrospun nanofibers (A) when hydrolyzed with 1.5 h of 0.1 M (B), 0.5 M (C) and 1 M (E) NaOH. Meanwhile, there was no observable difference between hydrolyzed PCL electrospun nanofibers treated with 1 h (D), 1.5 h (E) and 2 h (F) of 1 M NaOH. Similarly, the fiber diameter (G) and pore size (H) distributions of the elec-trospun PCL nanofibers at different durations of NaOH treatment shows no significant differences for the mean value ($p > 0.05$). n=47 for each duration in (G) and (H), respectively.

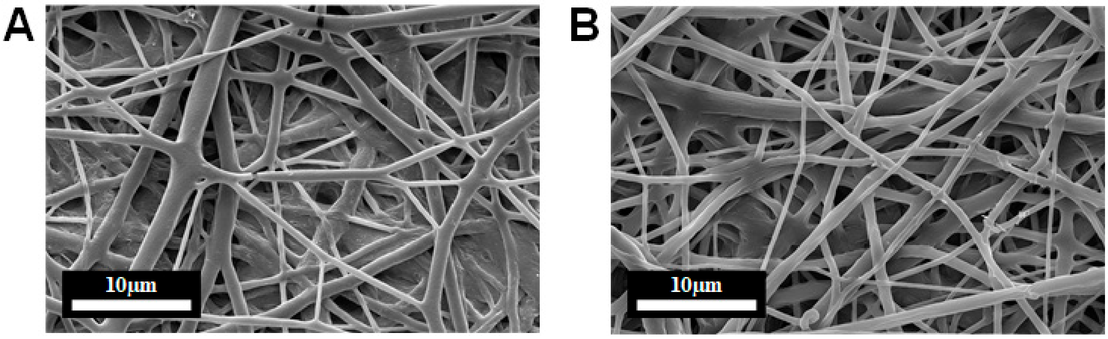

Figure S3. Effect of 70% ethanol soaking on the surface morphology of hydrolyzed electrospun PCL nanofibers. The hydrolyzed PCL electrospun nanofibers with 70% ethanol soaking (A) had a more flattened fiber structure and a more compact arrangement than the hydrolyzed electrospun PCL nanofibers without 70% ethanol soaking (B).

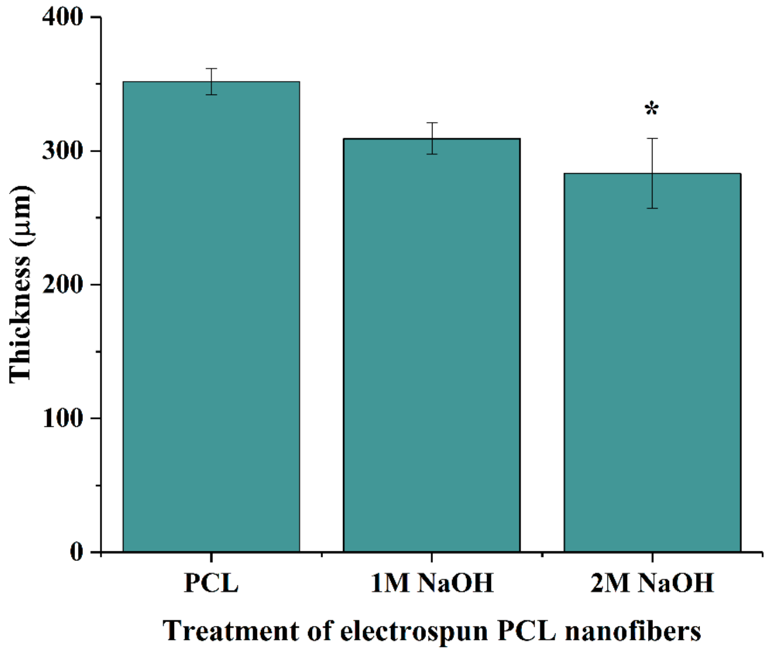

Figure S4. Thickness of the electrospun PCL nanofibers membrane after treatment of NaOH. The thickness of the nanofiber's membrane reduced significantly after 2 M NaOH treatment. Bars represent the mean and error bar represents the standard mean error of n=9. * represents significant difference compared with PCL ($p<0.05$, ANOVA with Tukey's post hoc test).

The measurements were based on optical microscope (Dino Light) readings of the membrane cross section view of samples fixed between two small pieces of magnets. All the samples used in this study were obtained after electrospinning of 20 ml of PCL solution and were selected to have relatively the same thickness. As shown in Figure S4, higher concentrations of NaOH has a destructive effect on the sample while lower concentrations can cause a slight reduction in sample thickness. The reduction is due to the peeling effect which has been mentioned in the manuscript.

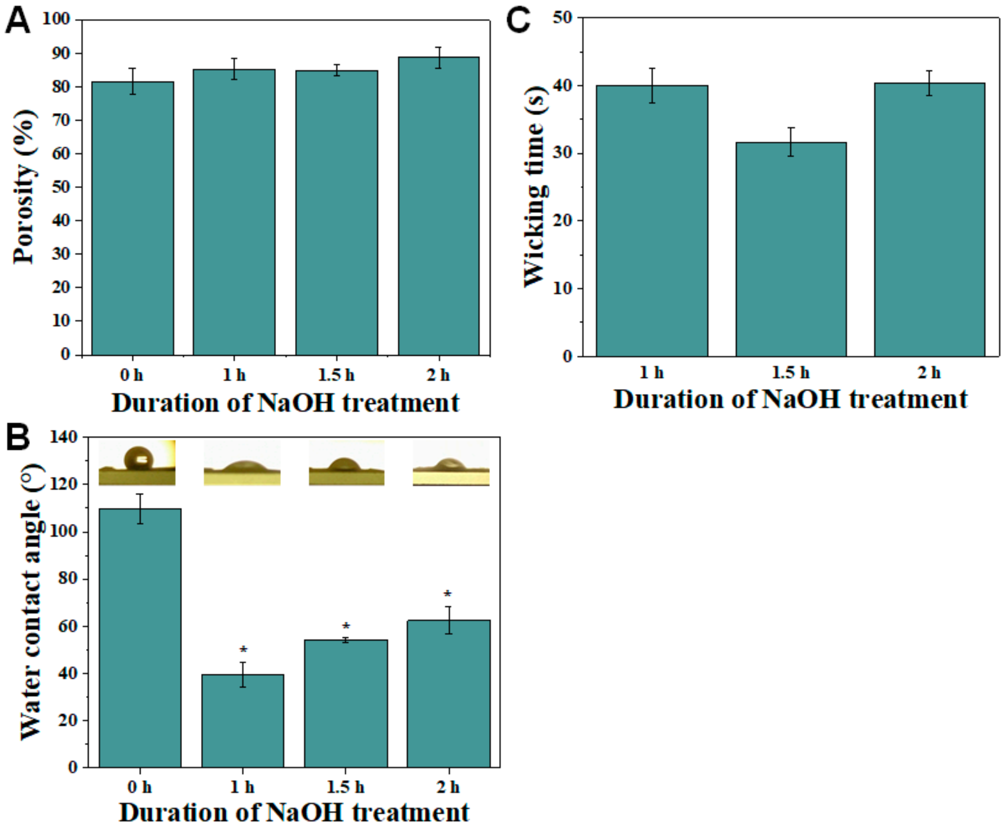

Figure S5. The influence of NaOH treatment duration upon wetting and wicking parameters. The porosity (A) of the post-treated nanofibers was not significantly affected by the duration of NaOH treatment. Notwithstanding, the water contact angle shows a significant reduction compared to non-treated membrane (0 h), yet no significant differences among the treatment durations (1 – 2 h) (B). The wicking time of liquid from sample pad to test line of NaOH-treated electrospun PCL nanofibres was not affected by the duration of the NaOH treatment (C). n=4 for each concentration in (A) and (B), and n=3 for each concentration in (C). * represents $p<0.05$.

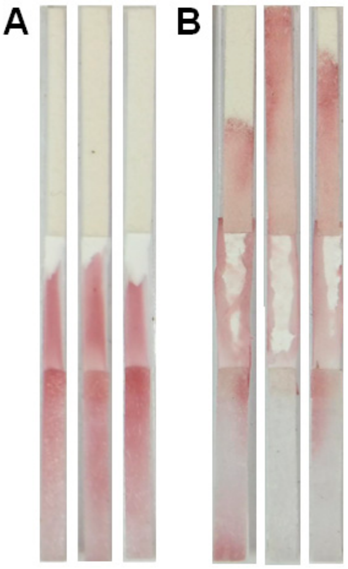

Figure S6. Wicking properties of the test strips with unoptimized hydrolyzed electrospun PCL nanofibers. LFA with PCL electrospun nanofibrous membrane hydrolyzed at 0.1 M NaOH did not achieve a full flow after 45 minutes of assay (A). Meanwhile, an LFA strip containing 1.5 h of 2 M NaOH treated PCL electrospun nanofibrous membranes without pre-soaking in 70% ethanol underwent non-uniform flow after 45 minutes of assay (B).

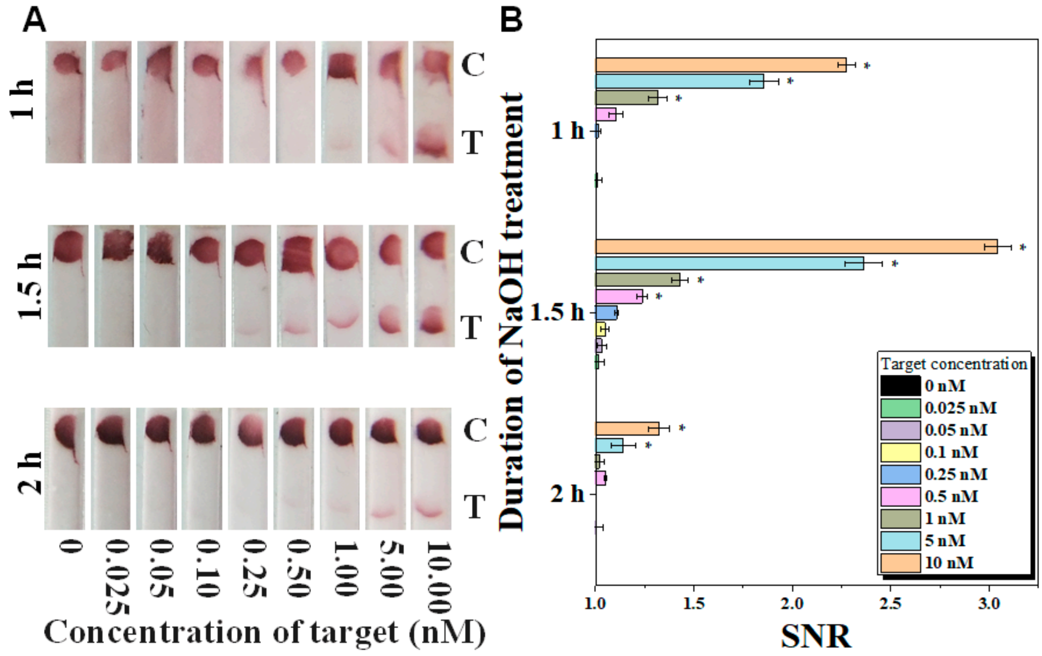

Figure S7. The effect of duration of NaOH treatment on the performance of lateral flow assay (LFA) integrated with hydrolyzed electrospun PCL nanofibers for the detection of zika viral DNA. Qualitatively, the background or noise signals were stronger at test strips with PCL electrospun membrane treated with NaOH concentration less than 1 h (A). The quantitative results for signal-to-noise ratio (SNR) showed the highest LOD at 0.5 nM for LFA with hydrolyzed PCL electrospun nanofibrous membrane treated with 1.5 h of 1 M NaOH (B). n=3. * represents $p<0.05$.

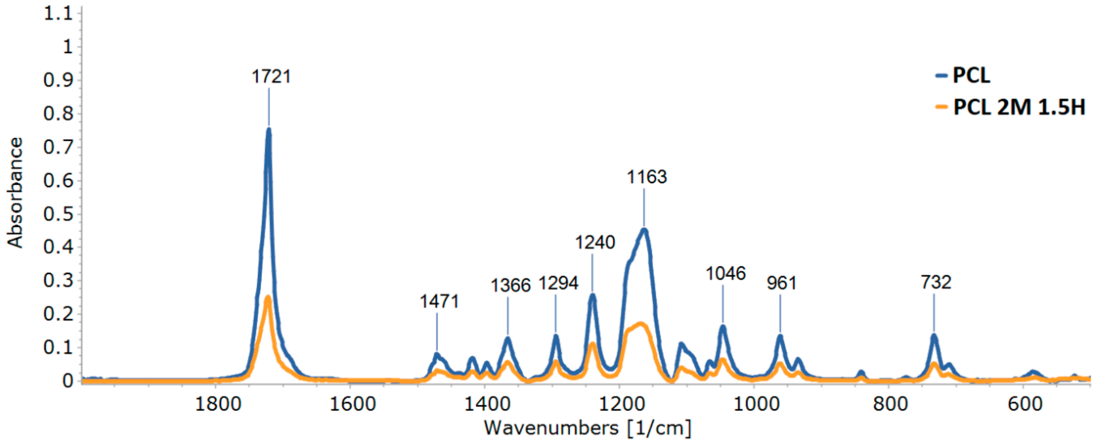

Figure S8. ATR- FTIR absorbance spectra of electrospun PCL plotted against electrospun PCL treated with 2M NaOH solution for 1.5 hour

A Perkin Elmer ATR-FTIR Spectrum 400 was used the collect the infrared spectra at a resolution ±2 cm^{-1} between 500 to 200 cm^{-}1. The peaks at 1721 and 1163 refer to carbonyl stretching and C-O and C-C stretching in amorphous phase, respectively [1]. The peak at 1294 corresponds to C-O and C-C stretching in the crystalline phase [2]. The significant reduction in intensity of the peaks is in line with thickness measurement and alkaline hydrolysis of PCL which was confirmed through water contact angle analysis.

References

1. Elzein, T.; Nasser-Eddine, M.; Delaite, C.; Bistac, S.; Dumas, P. FTIR study of polycaprolactone chain organization at interfaces. *Journal of colloid and interface science* **2004**, *273*, 381-387.
2. de Luca, A.C.; Terenghi, G.; Downes, S. Chemical surface modification of poly-ε-caprolactone improves Schwann cell proliferation for peripheral nerve repair. *Journal of tissue engineering and regenerative medicine* **2014**, *8*, 153-163.
